# The influence of high worry on static and dynamic insular functional connectivity

**DOI:** 10.3389/fnins.2023.1062947

**Published:** 2023-03-21

**Authors:** Youxue Zhang, Xueli Cai, Mingjun Duan, Hui He

**Affiliations:** ^1^School of Education and Psychology, Chengdu Normal University, Chengdu, China; ^2^The Clinical Hospital of Chengdu Brain Science Institute, MOE Key Lab for Neuroinformation, University of Electronic Science and Technology of China, Chengdu, China; ^3^Psychological Research and Counseling Center, Southwest Jiaotong University, Chengdu, Sichuan, China

**Keywords:** worry-proneness, interoception, insula, fMRI, dynamic functional connectivity

## Abstract

Worry is a form of repetitive negative thought. High worry-proneness is one risk factor leading to anxiety disorder. Several types of research indicated that anxiety disorder was highly associated with disrupted interoception. The insula is consistently considered to play a key role in interoception. However, the relationship between worry and the interoception network is poorly investigated in worry-prone individuals. Thus, it is essential to identify the neural characteristic of high worry-proneness subjects. A total of 32 high worry-proneness (HWP) subjects and 25 low worry-proneness (LWP) subjects were recruited and underwent magnetic resonance imaging scanning. Six subregions of insula were chosen as regions of interest. Then, seed-based static and dynamic functional connectivity were calculated. Increased static functional connectivity was observed between the ventral anterior insula and inferior parietal lobule in HWP compared to LWP. Decreased static functional connectivity was found between the left ventral anterior insula and the pregenual anterior cingulate cortex. Decreased dynamic functional connectivity was also shown between the right posterior insula and the inferior parietal lobule in HWP. Moreover, a *post-hoc* test exploring the effect of changed function within the insular region confirmed that a significant positive relationship between static functional connectivity (ventral anterior insula–inferior parietal lobule) and dynamic functional connectivity (posterior insula–inferior parietal lobule) in LWP but not in HWP. Our results might suggest that deficient insular function may be an essential factor related to high worry in healthy subjects.

## 1. Introduction

Worry is considered as one processing about a future event. Superabundant worry might have adverse effects on daily behavior (MacLeod et al., 1991). Based on DSM-IV-TR (Battle, 2013), the main features of a generalized anxiety disorder (GAD) are highly related to the process of worrying, such as chronic and excessive worry. Notably, high worry-proneness is more likely to develop into an anxiety disorder, such as GAD (Cox et al., 2022). Anxiety has a heavy burden on individuals and society (Kujanpää et al., 2017). Thus, it is essential to identify the neural characteristic of high worry-proneness subjects. Specifically, anxiety is not only characterized by worry but also accompanied by somatic symptoms (Cui et al., 2020). More important, several psychophysiological studies suggest that interoception plays a vital role in the interaction between internal function and cognitive processing (Chan et al., 2014, 2015). Thus, these findings consistently agree that anxiety disorder is fundamental to impaired interoception (Mallorquí-Bagué et al., 2016; Khalsa et al., 2018). In contrast, the underlying relationship between interception and worry is poorly investigated in worry-prone individuals.

Interoception refers to the nervous system’s processing of internal stimuli from its internal organs (e.g., heart, lungs, stomach, and bladder). This internal information would further affect subjects’ cognition and behavior (Van Oudenhove, 2010). Locating the substrates of interoception processes might provide more evidence to investigate the effects of high worry and anxiety on the human brain. Importantly, Badoud Deborah and Tsakiris Manos pointed out that the insula plays a key role during the interoception processes (Badoud and Tsakiris, 2017). The insula is a functional heterogeneous region with extensive connections to the cerebral cortex and limbic system. Thus, it implicates functions ranging from consciousness, interoceptive awareness, emotion processing, motor control, and interpersonal experience (Craig, 2009; Zaki et al., 2012). A neuroimaging study has begun to parcel the insular regions based on their patterns of functional mapping (Deen et al., 2011). Specifically, a study by Craig has indicated that an anatomical posterior–anterior progressive connectivity contributes to the integration between interoception and emotion, as well as cognition in the human brain (Craig, 2002), while the insular region and its potential relationship to interoception have not been measured in worry-proneness subjects.

Recently, resting-state functional magnetic resonance imaging (rs-fMRI) has been commonly used to measure the inter-regional static functional connectivity (FC) in the human brain. Specifically, the developments of dynamic functional connectivity (dFC) analyses have documented that the FC is changing over time. Moreover, the dFC could sensitively capture the deficient dynamic feature associated with psychiatric symptoms (Ma et al., 2014; Du et al., 2016). A recent study also indicated that the feature of dFC significantly outperforms the static FC through the classification analysis (Rashid et al., 2016). The static FC might obscure the dynamic features of network behavior. Thus, these findings posit that static and dynamic FC could provide complementary information about the neural substrates of worry-proneness and anxiety disorder, while, in recent years, there has been no study exploring the interoception functional network in worry-proneness subjects through static and dynamic FC analyses.

The current study was conducted to investigate the deficient insular functional network in a sample of worry-prone individuals. To this end, participants were subjected to rs-fMRI. Based on the results from previous behavioral and neuroimaging research, we hypothesized that the differences in insular functional connectivity might be found in high worry-prone individuals. We posit that these changed insular functions may be associated with worry in healthy subjects.

## 2. Materials and methods

### 2.1. Demographic characteristics

A total of 57 college students were selected from an initial sample of 1,224 students who completed the Penn State Worry Questionnaire (PSWQ) (Zhong et al., 2009). Thirty-two subjects who scored within the top one-tenth (10%) of the PSWQ distribution (range 55–76) were clarified into a high worry-proneness (HWP) group. Low worry-proneness (LWP) subjects were 25 students who scored within the bottom one-tenth (10%) of the distribution (range 19–37). The study was approved by the Ethics Committee of the Clinical Hospital of Chengdu Brain Science Institute following the Helsinki Declaration (IRB number: 2020036). Informed consent was obtained from all subjects included in the study.

### 2.2. Data acquisition

Imaging was acquired on a 3 T MRI scanner (GE DISCOVERY MR750) at the University of Electronic Science and Technology of China. First, high-resolution T1-weighted images were acquired using a three-dimensional fast spoiled gradient-echo sequence [repetition time (TR) = 6.008 ms, flip angle (FA) = 9°, matrix = 256 × 256, field of view (FOV) = 256 × 256 mm^2^, slice thickness = 1 mm, no gap, 152 slices]. Second, rs-fMRI data were acquired using gradient-echo echo planar imaging sequences [TR = 2,000 ms, echo time (TE) = 30 ms, FA = 90°, matrix = 64 × 64, FOV = 240 × 240 mm^2^, slice thickness/gap = 4 mm/0.4 mm, number of slices = 42], with an eight channel-phased array head coil. Approximately 255 volumes were obtained. During the rs-fMRI scan, subjects were instructed to have their eyes closed.

### 2.3. rs-fMRI preprocessing

Functional image preprocessing was performed using Neuroscience Information Toolbox (NIT) (Dong et al., 2018) according to a standard pipeline as follows: (1) Slice time and head motion correction, normalization (3 mm * 3 mm * 3 mm) into TPM template and image smoothing (FWHM 6 mm). (2) Detrending analysis was performed. (3) Temporal filtering (bandpass 0.01–0.08 Hz). (4) The nuisance signals were regressed, including six motion parameters and their first temporal derivative, white matter signal, and cerebrospinal fluid signal. The global signal was not regressed (Yang et al., 2014). (5) Framewise displacement (FD) was evaluated for all subjects as suggested by Power et al. (Power et al., 2012). The FD score of each subject was calculated by using the following formula:


$$\begin{array}{l} FD=\\ \frac{1}{M-1}\sum_{i=2}^{M}\sqrt{{\left|\Delta t_{x_{i}}\right|}^{2}+{\left|\Delta t_{y_{i}}\right|}^{2}+{\left|\Delta t_{z_{i}}\right|}^{2}+{\left|\Delta d_{x_{i}}\right|}^{2}+{\left|\Delta d_{y_{i}}\right|}^{2}+{\left|\Delta d_{z_{i}}\right|}^{2}} \end{array}$$

where M is the length of time courses, and x_i_, y_i_, and z_i_ are translations/rotations at *ith* time point in the x, y, and z directions; similar to $\Delta t_{y_{i}}$ and $\Delta t_{z_{i}}$, ∆t represents the FD rotations and ∆d represents the FD translations. Then, we also examined the group-level FD difference between the two groups using a two-sample *t-test*. Moreover, subjects who had a maximum translation in any orthogonal direction larger than 3 mm or rotation larger than 3 degrees were excluded from subsequent analysis.

### 2.4. Static and dynamic functional connectivity analyses

The six seeds were chosen, including left and right ventral anterior insula (vAI), left and right dorsal anterior insula (dAI), and left and right posterior insula (PI) (Deen et al., 2011). Then, the static functional network was calculated. The Pearson correlation coefficients were defined as static FC. The resulting values were transformed through Fisher’s r-to-z transformation. Furthermore, sliding-window-based dynamic FC was performed between the seed and all voxels in the brain as follows: (1) The time courses were segmented into 100-s (50TR) windows (*f_min_* = 0.01 Hz), sliding the onset by 2-s (1TR) (Leonardi and Van De Ville, 2015). (2) The Pearson correlation coefficient was calculated between the seed and all voxels within each window. (3) The standard deviation (SD) of correlation coefficients across the window was defined as a dynamic FC score.

### 2.5. Statistical analysis

Age and gender were regressed as potential confounding covariates from static and dynamic FC mappings. Then, a two-sample *t-test* was used to assess the different functional connectivity between the two groups. Both Bonferroni and AlphaSim corrections were used for the 12 comparisons. First, the correction threshold of each comparison is calculated using Bonferroni correction with *p* < 0.05. Thus, the resulted score is *p* < 0.004 (0.05/12). Second, the voxel-wise comparison was corrected within each FC map using AlphaSim correction at *p* < 0.004 (combination of a threshold of voxel-level *p* < 0.001 and a minimum cluster size threshold) through REST software.^1^

### 2.6. Correlations with worry-proneness factor

To measure the relationship between the altered functional connectivity and worry-proneness feature, we extract the mean functional score from the peak voxel and its nearest voxels (26 voxels) for each significant cluster. Then, the partial correlation analysis was performed between the mean changed static/dynamic FC of the worry-proneness group and the score of PSWQ with age, gender, and FD score as covariates in each group. Moreover, the association between the two groups was calculated using permutation testing. Using the low worry-proneness as a baseline, a negative difference between groups implied a relative loss of the relationship. In contrast, the positive differences might reflect a compensatory mechanism.

### 2.7. Validation analysis

To ensure a high reproducibility of our statistical results, the validation analyses were performed as follows: (1) The permutation testing was used to assess the difference in abnormal static and dynamic FC of HWP compared to LWP, and (2) 25 subjects were randomly selected from HWP. Then, the two-sample *t-test* was used to assess the different static and dynamic functional connectivity between selected HWP and LWP subjects. These steps were repetitively calculated 200 times. Then, we showed the probability map of 200 difference maps where the voxels exhibited significant group differences (AlphaSim correction at *p* < 0.004).

## 3. Results

### 3.1. Demographic and clinical data

No subject was excluded because of excessive head motion. There is no significant difference between the two groups in terms of age and gender (Table 1).

**Table 1:** Participant fundamental information.

	High worry-proneness group	Low worry-proneness group	*p*
Gender (Male/Female)	18/14	12/13	0.383^a^
Age (years)	21.06 ± 1.95	21.76 ± 1.89	0.181^b^
PSWQ score	63.03 ± 5.75	32.08 ± 4.39	<0.001^b^
FD	0.14 ± 0.03	0.14 ± 0.02	0.871^a^

Indicated values are shown as mean ± standard deviation. PSWQ: Penn State Worry Questionnaire. FD: Framewise displacement.

a indicates the *p*-values for the comparisons (chi-square test) between two groups.

b indicates the *p*-values for the comparisons (two-sample t-tests) between two groups.

### 3.2. Altered static and dynamic functional connectivity

**Figure 1:**
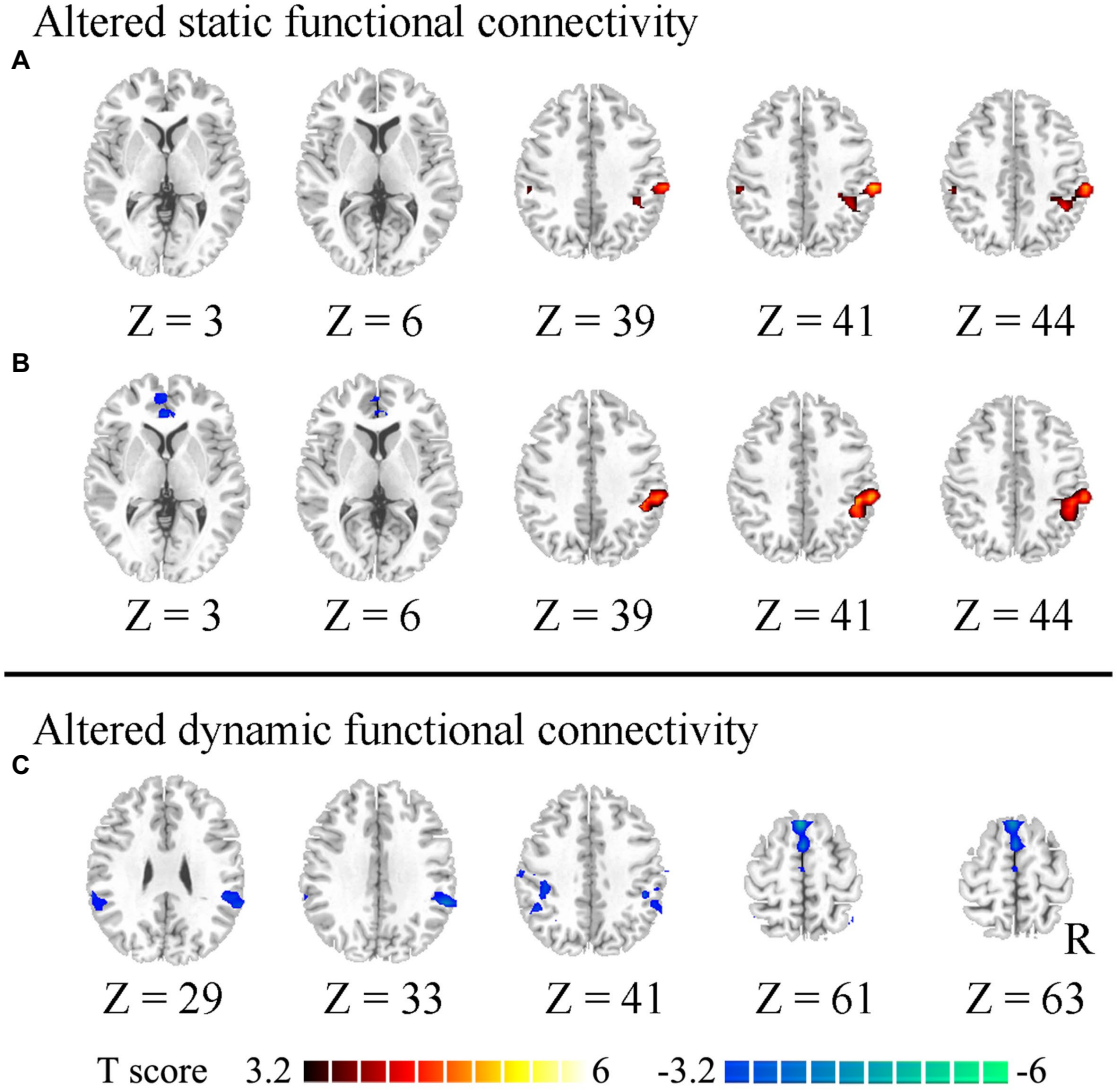
Altered static and dynamic functional connectivity (FC) of insular subregions. **(A)** indicates increased static FC between the right ventral insula and right temporoparietal junction (TPJ) in the high worry-proneness group compared to the low worry-proneness group. **(B)** indicates increased static FC between the left ventral insula and right TPJ and decreased static FC between the left ventral insula and pregenual anterior cingulate cortex in the high worry-proneness group. **(C)** indicates decreased dynamic FC between the right posterior insula and TPJ, as well as supplementary motor area in the high worry-proneness group.

**Figure 2:**
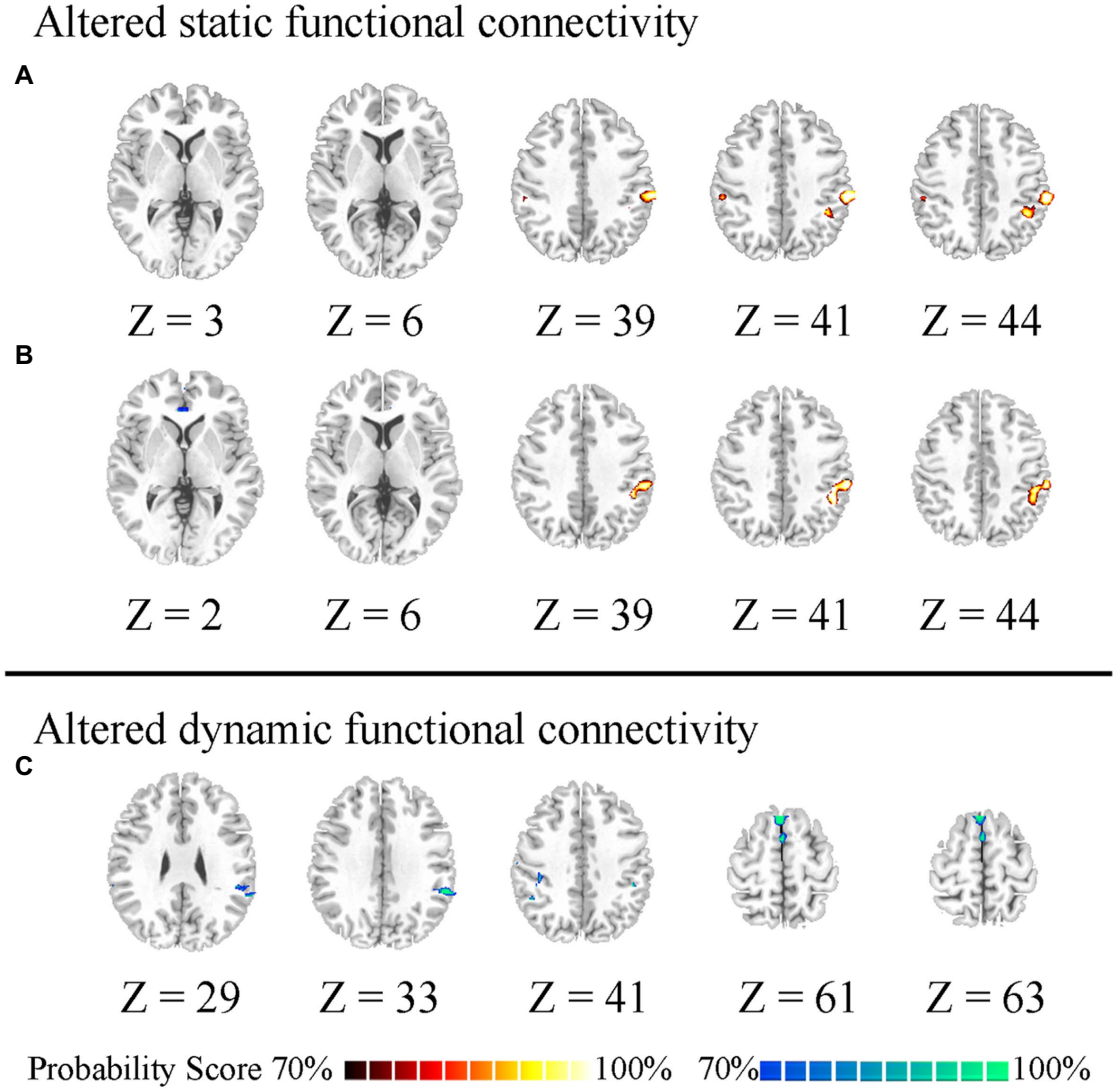
Probability difference map. “70%” indicates the percentage of significant difference of 200 statistical analyses between the part of the high worry-proneness group (HWP) and low worry-proneness group (LWP). **(A)** indicates increased static FC between the right ventral insula and right temporoparietal junction (TPJ) in HWP. **(B)** indicates increased static FC between the left ventral insula and right TPJ and decreased static FC between the left ventral insula and pregenual anterior cingulate cortex in HWP. **(C)** indicates decreased dynamic FC between the right posterior insula and right TPJ, as well as supplementary motor area in HWP.

The static and dynamic FC patterns of the insular subregion were similar across the two groups. First, within the comparison of static FC, increased FC was found between the right vAI and right temporoparietal junction (TPJ) in HWP compared to LWP (Figure 1A). The HWP also showed increased static FC between left vAI and right TPJ. Moreover, decreased static FC was observed between left vAI and pregenual anterior cingulate cortex (PgACC) (Figure 1B). Second, within dynamic FC, the decreased dynamic FC was found between right PI and TPJ, as well as SMA in HWP (Figure 1C). These changed static and dynamic FCs were not associated with age, gender, and FD scores, respectively. Furthermore, the nearly consistence changes in static and dynamic FC were observed in HWP group compared to LWP group through permutation testing. Similar results were also observed between the statistical finding of all subjects and the probability map of part of the subjects (Figure 2).

**Figure 3:**
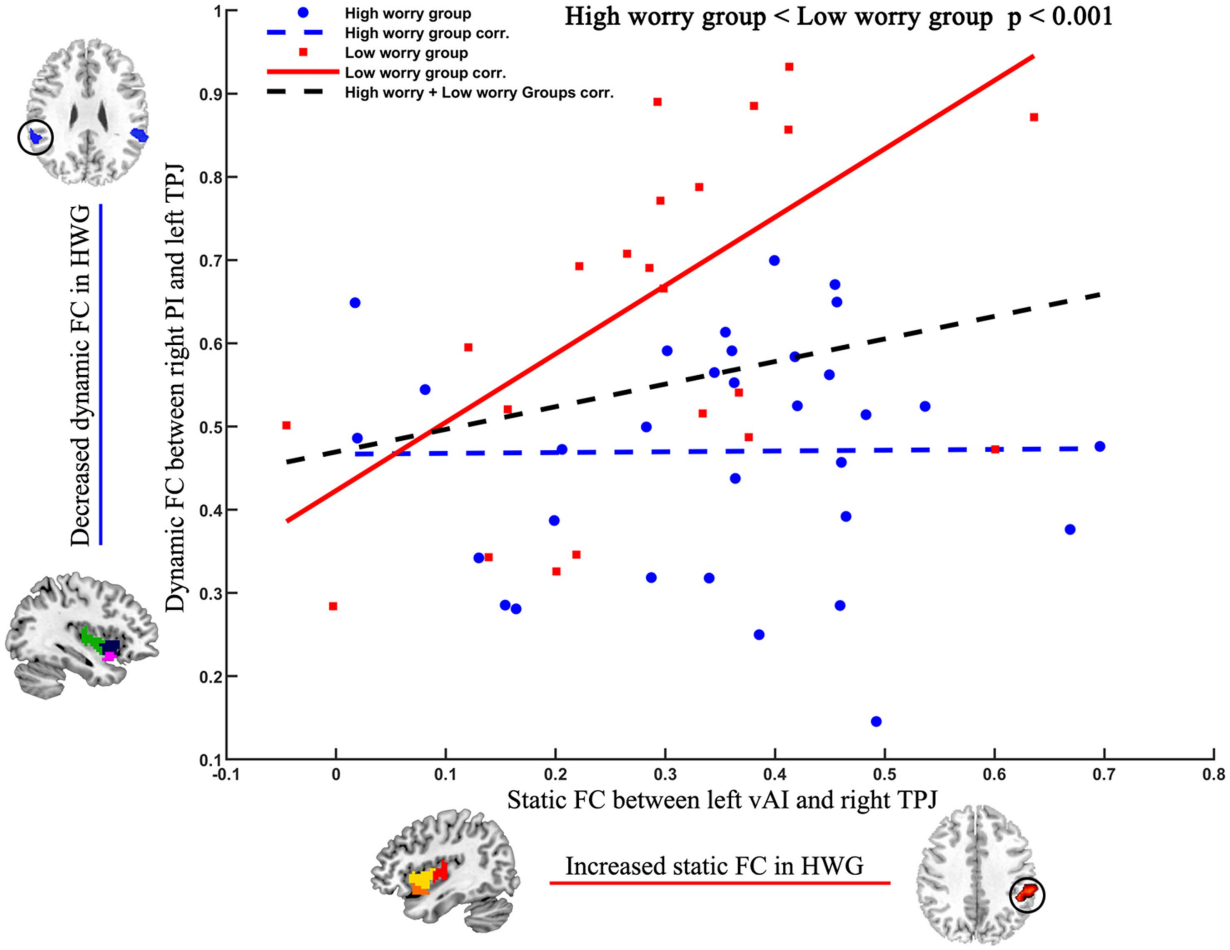
Different relationships between different FC of the posterior insula and changed FC of the anterior insula in two groups. Positive relationship (*r* = 0.5728, *p* = 0.0028) between static FC (left vAI–right TPJ) and dynamic FC (right PI–left TPJ) in the low worry-proneness group (red node and line). Whereas this correlation (*r* = 0.0113, *p* = 0.9512) was not apparent in the high worry-proneness group (blue node and line), the difference between these two correlation coefficients was significant (*p* < 0.001).

As a *post-hoc* analysis to determine the relationship between different FC of the posterior insula and changed FC of the anterior insula, in the low worry-proneness group, we observed a positive relationship (*r* = 0.5728, *p* = 0.0028) between static FC (left vAI–right TPJ) and dynamic FC (right PI–left TPJ) (Figure 3), whereas this correlation was not apparent in the high worry-proneness group (*r* = 0.0113, *p* = 0.9512). The difference between these two relationships was also significant (*p* < 0.001), suggesting that the coupling of the posterior insula and TPJ may be associated with the FC between the anterior insula and TPJ in low worry-prone individuals (Table 2).

**Table 2:** Significant static and dynamic functional connectivity.

Regions	MNI coordinates			T-score	Cluster voxel
	*x*	*y*	*z*		
Static FC with right vAI					
TPJ.R	56	−30	41	5.375	52
Static FC with left vAI					
TPJ.R	54	−31	41	5.053	82
PgACC	−2	36	3	−3.779	37
Dynamic FC with right PI					
SMA	−5	23	61	−5.011	79
TPJ.R	56	−40	29	−4.397	68
TPJ.L	−61	−42	29	−3.831	34

FC: functional connectivity; vAI: ventral anterior insula; IPL.R: right inferior parietal lobule; PI: posterior insula; PgACC: pregenual anterior cingulate cortex; TPJ: temporoparietal junction; SMA: supplementary motor area.

### 3.3. Relationship between altered FC and PSWQ score

**Figure 4:**
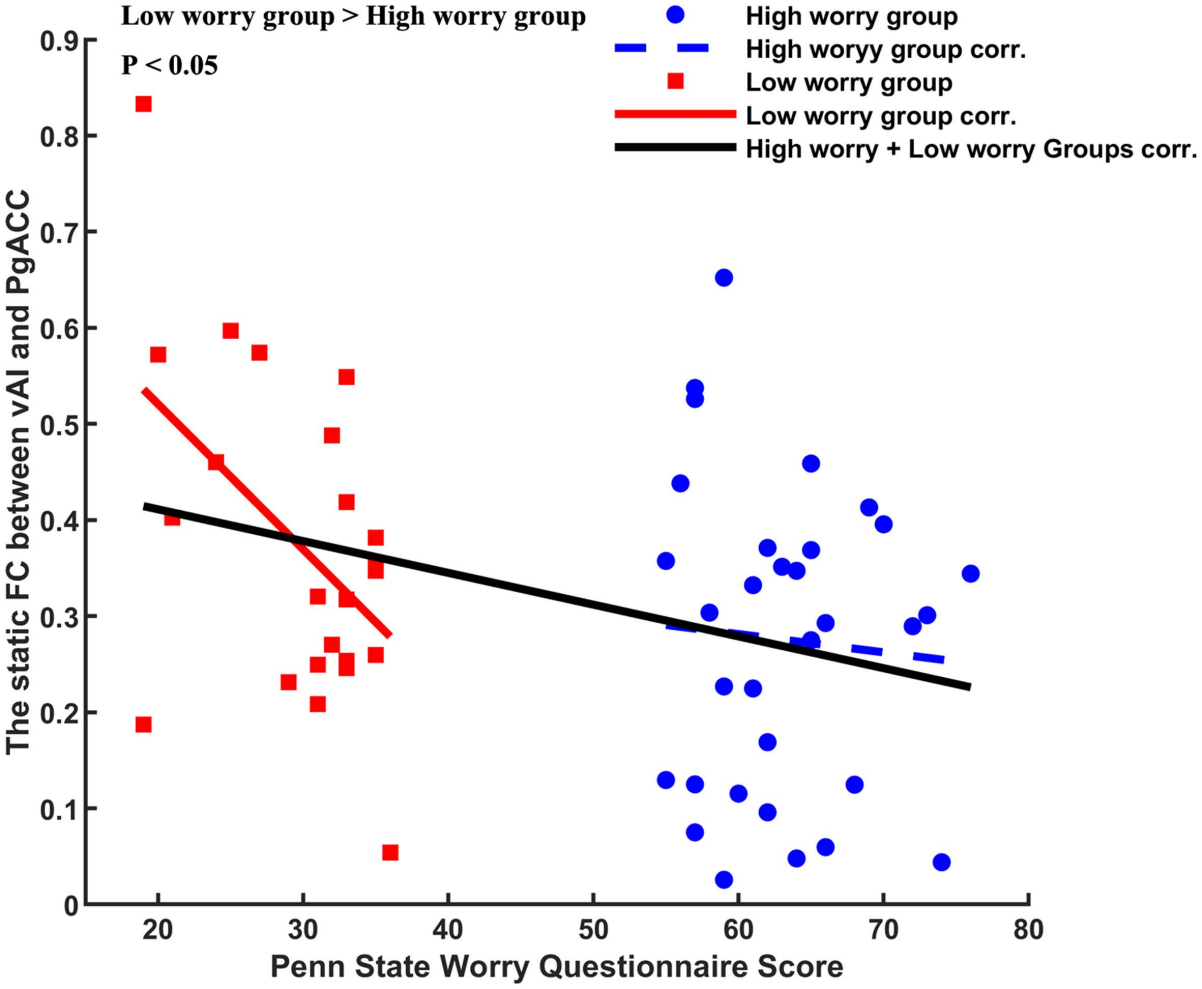
Different correlations between Penn State Worry Questionnaire (PSWQ) score and altered static left vAL-PgACC (pregenual anterior cingulate cortex). Negative relationship (*r* = −0.5000, *p* = 0.0109) was found in the low worry-proneness group (red node and line), whereas this correlation (*r* = −0.0677, *p* = 0.7127) was not apparent in the high worry-proneness group (blue node and line). The difference between these two correlation coefficients was significant (*p* < 0.05).

We observed a negative correlation between the PSWQ score and static FC (left vAI and PgACC) in the low worry-proneness group (*r* = −0.5000, *p* = 0.0109), whereas this correlation was not apparent in the high worry-proneness group (*r* = −0.0677, *p* = 0.7127). The difference between these two correlation coefficients was also significant (*p* < 0.05) (Figure 4).

## 4. Discussion

Anxiety disorder would lead to significant personal and economic burdens. Exploring the change in the brain before the occurrence of the disease has great significance for the prevention and cure of anxiety disorder. In this study, we investigated the abnormal neural substrates of the insula in a sample of individuals with worry-proneness. The decreased FC of insula–ACC and increased insula–TPJ were observed in HWP compared to LWP, respectively. These changed FCs might reflect a remodeling of the insular function network with high worry.

Patients with GAD have high body perception scores than healthy controls (Ehlers and Breuer, 1992; Cui et al., 2020). Higher body perception scores reflected hypersensitive interoception in anxiety disorder. In the human brain, the posterior insula plays a crucial role in integrating the physical signal of interoception (Kuehn et al., 2016). Information is initially transferred to the posterior insula from the individual’s inner organs (e.g., heart rate) and self-generated movements (Limanowski et al., 2014). During the somatosensory integration, the posterior insula gate this signal (Palmer et al., 2016). Moreover, Jakub Limanowski’s research reported that the gating processing would extend to SMA from the posterior insula (Limanowski et al., 2020). These gating might underlie the brain’s capacity to attenuate self-generated somatosensory reafference. In this study, the decreased dynamic PI-TPJ and PI-SMA FCs were found in HWP. TPJ is located at the ventral–anterior section of the inferior parietal lobule and surrounds the posterior end of the Sylvian fissure. Specifically, the ventral intraparietal region is associated with multi-modal representations, such as visual, auditory, and somatosensory information (Avillac et al., 2005; Schlack et al., 2005). Decreased dynamic PI-TPJ/SMA FCs might induce deficient gating processing of PI. Thus, our results indicated that the high worry might be associated with the abnormal dynamic PI-TPJ/SMA FCs in the human brain.

In addition to the connectivity between somatosensory cortices and posterior insula, cerebral substrates of interoception were consistently defined as the critical pathway, including the anterior insular region and anterior cingulate cortex (Craig, 2009; Craig, 2010). Importantly, these two pathways have been highly linked through anatomical connectivity with the insula. More important, one hypothesis of pathological mechanisms of mental disorder is that interoceptive input (i.e., posterior insula) becomes increasingly decoupled from the self-generated somatosensory region, leading to abnormal interoceptive prediction error (Bonaz et al., 2021; Weng et al., 2021). In this study, the increased static FC was observed between the anterior insula and TPJ. Moreover, a positive relationship was observed between increased static FC (anterior insula and TPJ) and decreased dynamic FC (posterior insula and TPJ) in LWP, whereas this correlation was not found in HWP. TPJ is one key region within the salience network and has highly functional connectivity with the anterior insula (Onofrj et al., 2022). Consistent with previous studies, our findings indicated that interoception processing might be associated with the function coupling between the anterior insula and posterior insula. Furthermore, our results revealed that the high worry might be associated with the deficient decoupling functional connectivity between the posterior insula and anterior insula through TPJ.

The anterior insula plays a central role in integrating multi-interoceptive physical features with emotional salience (Menon and Uddin, 2010; Kuehn et al., 2016; Sturm et al., 2018). Ventral insula functional connectivity with limbic and autonomic processing regions (e.g., PgACC, thalamus, and amygdala) makes up the salience network (Pasquini et al., 2020), which represents the homeostatic significance of prevailing stimuli and inner conditions. Specifically, the functional connectivity between the anterior insula and anterior cingulate cortex mainly contributes to the dorsal lateral prefrontal cortex (dlPFC)-involved cognitive control triggered by salience evaluation (Sridharan et al., 2008). Moreover, previous research indicated that high trait anxiety and GAD share common changed FC of the salience network (Etkin and Wager, 2007; Sylvester et al., 2012). Adolescents’ trait anxiety score was negatively related to FC between the anterior insula and anterior cingulate cortex (Geng et al., 2015). Thus, worry or anxiety might be negatively related to the FC between the two regions. In this study, the decreased static FC was observed between the left ventral insula and PgACC in high worry-proneness subjects compared to low worry-proneness subjects. Our findings indicated that the deficient static anterior insula–PgACC FC might be related to the effect of worry on healthy subjects. The findings of the changed function of the anterior insula in HWP might be of great significance to understanding the pathological mechanisms of anxiety disorder.

Several limitation issues need to be considered in this study. First, the seed-based FC analysis was performed. The regions of interest were defined as hypothesis-driven, which might have disregarded results. However, it is easy to replicate our findings. Second, the somatosensory and interoceptive functions were not acquired from the participants. Our results should be validated and expanded in further study. Finally, our results were observed in HWP, and this result should be validated and extended to anxiety disorder.

## 5. Conclusion

In conclusion, our results reveal the altered static and dynamic insular function network in HWP subjects through resting-state static and dynamic FC approaches. We show the functional decoupling between the anterior and posterior insular subregions in the HWP group. Combined with deficient static vAI-PgACC FC, our findings highlight that the remodeling of the insular function network might contribute to the effect of high worry on healthy subjects.

## Data availability statement

The raw data supporting the conclusions of this article will be made available by the authors, without undue reservation.

## Ethics statement

The studies involving human participants were reviewed and approved by the Ethics Committee of The Clinical Hospital of Chengdu Brain Science Institute following the Helsinki Declaration (IRB number: 2020036). The patients/participants provided their written informed consent to participate in this study.

## Author contributions

YZ, MD, and HH had made a substantial contribution to the conception and drafting and revising the article. XC acquired the data. YZ and HH had made a substantial contribution to the analysis and interpretation of the data. All authors contributed to the article and approved the submitted version.

## Funding

This study was partly supported by a grant from the National Nature Science Foundation of China (Grant numbers: 62003076, 81801775), the Chengdu Science and Technology Bureau (Grant number: 2021-YF09-00107-SN), the Project of Natural Science Foundation of Sichuan Province (2023NSFSC1489 and 2023NSFSC1186), and the Special Project of Sichuan Postdoctoral Foundation (TB2022089).

## Conflict of interest

The authors declare that the research was conducted in the absence of any commercial or financial relationships that could be construed as a potential conflict of interest.

## Publisher’s note

All claims expressed in this article are solely those of the authors and do not necessarily represent those of their affiliated organizations, or those of the publisher, the editors and the reviewers. Any product that may be evaluated in this article, or claim that may be made by its manufacturer, is not guaranteed or endorsed by the publisher.

## Footnotes

^1^ http://www.restfmri.net
